# Effect of Auricular Acupressure on Acute Pain in Nursing Home Residents with Mild Dementia: A Single-Blind, Randomized, Sham-Controlled Study

**DOI:** 10.1155/2022/6406383

**Published:** 2022-03-10

**Authors:** Jun-Jun Zhang, Li Yu, Jun-Hui Mei, Hong-Xin Wang, Hai-Xiang Gao, Ju-Fang Fu, Ye Cheng, Lu-Lu Gao, Lei Bu, Jian-Qiang Yu, Carol Chunfeng Wang, Yu-Xiang Li

**Affiliations:** ^1^Department of Hematology and Oncology, International Cancer Center, Shenzhen Key Laboratory, Shenzhen University General Hospital, Shenzhen University Clinical Medical Academy, Shenzhen University Health Science Center, Xueyuan Avenue 1098, Shenzhen 518000, China; ^2^School of Nursing, Ningxia Medical University, 1160 Sheng Li Street, Yinchuan, China; ^3^Department of Emergency, Yinchuan Second People's Hospital, 684 Beijing Road, Yinchuan, China; ^4^Department of Nursing, Shenzhen University General Hospital, 1098 Xueyuan Avenue, Shenzhen, China; ^5^School of Public Health, Xinxiang Medical University, 601 Jinsui Avenue, Xinxiang, China; ^6^Department of Foreign Language Teaching, Ningxia Medical University, 1160 Sheng Li Street, Yinchuan, China; ^7^Department of Pharmacology, Pharmaceutical Institute of Ningxia Medical University, 1160 Sheng Li Street, Yinchuan, China; ^8^School of Nursing and Midwifery, Edith Cowan University, 270 Joondalup Dr, Joondalup, WA 6027, Australia

## Abstract

**Introduction:**

Acute pain is a prevalent problem for dementia residents in nursing homes. A variety of intervention strategies have been applied to address this problem. However, there remains an issue of inadequate pain control. This study aims to explore the analgesic efficacy of auricular acupressure (AA) for dementia residents with acute pain in nursing homes.

**Methods:**

A multicenter, single-blind, randomized, and sham-controlled clinical trial was performed in three nursing homes in Yinchuan, China. All of the 206 eligible patients with acute pain were randomly divided into two groups for real AA therapy or sham AA (at sham point stimulation) therapy. The primary outcome was measured with a face pain scale revised (FPS-R) score before the procedure, 5 min after the start of the intervention, and 5 min after finishing the procedure. Secondary outcomes covered three physiological parameters, adverse reactions observed, satisfaction level of caregivers, acceptance of patients, and additional use of analgesics.

**Results:**

There was a significant difference in pain scores based on FPS-R between the two groups (*p* < 0.01). Pain score in the true AA group was 1.84 ± 0.23, compared with 2.22 ± 0.81 in the sham AA group. No adverse events were found during the whole procedure for all patients. The satisfaction level of caregivers and acceptance of patients in the real AA group were significantly higher than those in the sham AA group.

**Conclusion:**

This study shows that real AA was an alternative analgesic modality in reducing acute pain in patients with mild dementia.

## 1. Introduction

It has been estimated that there are over 50 million patients with dementia around the world, and over 20% of them are in China [1, 2]. By 2030, cases of dementia in China are expected to over 16 million at the current rate of growth [2]. As expected, up to 80% of residents living in nursing home settings have a diagnosis of dementia [3].

Pain is the most common complaint of residents with dementia in nursing homes. The prevalence of pain among residents is significantly higher than in other communities, with numerous studies indicating that as many as 40% to 80% of these residents with dementia were affected by pain [4–6]. A study in Europe reported that up to 82.9% of senior citizens with dementia experience pain in the nursing home [7]. Pain is not only an unpleasant subjective experience but also the main culprit of many detrimental consequences. They include neuropsychiatric symptoms, social interaction impairment, poor quality of life, lack of appetite, and other geriatric syndromes and events, including suicide attempts [7–9]. Potentially, the risk of disability increases with pain intensity [9].

These guidelines recommend paracetamol as a first-line approach for dementia patients with communication problems, which aligns with systematic reviews [10–12]. Currently, it is used in nursing homes worldwide. Acute or long-term overdose of paracetamol can contribute to hepatotoxicity, especially for the elderly who are more susceptible to drug-related adverse effects [8]. Patients with dementia are facing more challenges than those without cognitive impairment regarding controlling pain. The amount of analgesics prescribed by the doctor is only one-third of the normal cognitive elderly, which does not have access to the best clinical standard [13]. The use of strong opioids for dementia patients with moderate to severe pain is appropriate in some national guidelines [8, 11], but they receive fewer opioids because medical workers, patients, and family members are concerned about the potential negative side effects [13, 14]. Studies have shown that pain management may be insufﬁcient or inappropriate in nursing home settings [8, 10]. Efforts to explore non-pharmacological therapies to control pain have never stopped. There are also guidelines that emphasize the importance of non-pharmaceutical strategies [15, 16]. And some studies report that non-pharmaceutical measures are effective in controlling pain and reducing opioid use [15, 17]. Therefore, it is important to seek non-drug therapies for treating dementia patients with acute pain. Traditional Chinese medicine, such as auricular acupressure, acupuncture, massage, liniments, ointments, and so on, plays an active important role in the treatment of acute pain. In traditional Chinese medicine (TCM) theory, the ear is where the body's meridians (main twelve meridians: six of which are yin meridians and six yang) connect and the essential Qi converges. Therefore, stimulating auricular points can activate Qi and blood circulation, dredge the meridians, and then achieve the purpose of analgesia [18].

The use and safety of auricular acupressure (AA) have been well studied in managing acute postoperative pain after surgery, primary dysmenorrhea, and other medical conditions [17, 19, 20]. This study aims to verify the hypothesis that AA can produce analgesic effects on dementia patients with acute pain in nursing homes.

## 2. Materials and Methods

### 2.1. Study Design

This study is a multicenter, single-blind, randomized, and sham-controlled clinical trial of AA as described in a study protocol [21]. The trial is performed from April 2018 to March 2020 at old-age care institutions in Yinchuan, China. The study has been approved by Ningxia Medical University Ethics Committee (2018–232). The trial has been registered at the Chinese Clinical Trial Registry (No.: ChiCTR1800019146). Written informed consent was obtained from all subjects and guardians of them in this study.

### 2.2. Participants

Nursing home residents who were recruited into the study must meet the following standards: 60 years or older, mild dementia (according to the Chinese Mini-Mental State Examination, CMMSE) [22], the score of acute pain ≥4 (based on the face pain scale revised, FPS-R), able to access the multifunctional pulse oxygen monitor, and sign the informed consent for participating. Exclusion criteria included allergic history to adhesive tapes and alcohol, experience with AA before this study, moderate or severe dementia, acute abdomen, medical contraindications (ear inflammation, ulcer, and frostbite), severe heart, brain, liver, kidney, or hematopoietic diseases.

### 2.3. Treatments

Patients eligible for recruitment were randomly assigned to an intervention or a control group. The intervention group received routine pain management plus real AA therapy (medical adhesive tape with raw Semen Vaccariae), while routine pain management plus sham AA therapy (at sham points stimulation) was applied to the control group. Auricular-plaster therapy was an adhesive tape with a seed in the middle fixed to various auricular points, stimulating the auricular points with your fingers on both ears. There were significant differences in terms of the selected acupoints and function of acupoint combination between the two groups. In this study, Shenmen (TF4), Subcortex (AT4), Adrenal gland (TG2P), and two anatomical regions corresponding to the site of pain were selected to relieve pain in the intervention group. These five points (kidney, CO10; ocuiput, AT3; internal ear, L06; external ear, TG1u, and adrenal gland, TG2P) were selected in the control group. The main effect of these five points was to delay hearing loss. Two anatomical regions were selected by the inverted fetus map [23]. Prior to the intervention, a professionally trained practitioner used an auricular point detector (XS-100A; Guangzhou City Strong Medical Technology Co. Ltd., Guangzhou, China) to find the sensitive points, which are the target acupoints. It is used to probe the corresponding ear area gradually from the periphery to the center or probe the positive reaction points. The pressure should be light, slow, and uniform. Target points would be disinfected using 75% alcohol. Then, adhesive tapes (6 mm × 6 mm) were attached to the selected acupoints on both ears. Then, the practitioner pressed continuously the acupoints for 1 min per point 2 times. The whole intervention lasted for 10 minutes. The optimal pressure was considered as that patient felt localized tingling pain, warm sensation, and/or numbness and within the patient's tolerance. For the control group, the number and frequency of pressing were the same as the intervention group besides the selection of auricular acupoints. After the intervention, tapes were removed from the ears [24]. Optimal pressure was that patients felt localized tingling pain, warm sensation, and/or numbness and within the patient's tolerance.

### 2.4. Binding

All of the 206 patients deemed eligible were randomized into the real AA group or the sham AA group by using a randomization list. Patients were assigned to two groups at 1:1 ratio. The randomization list was generated by an independent statistician with the random number generator in SPSS 22.0 (SPSS Inc., Chicago, IL, USA). And only the project manager and operator had access to it. It was blinded for other researchers (data collectors and administrators, outcome assessors, statisticians, etc.) as well as patients and medical workers to the subject's sequence number throughout the whole trial. To reduce bias, researchers were not allowed to communicate regarding data collection.

### 2.5. Sample Size

Prior to the study, we conducted a pilot trial with a sample size of 30 patients in a nursing home in Yinchuan. The change of pain score was the primary outcome. The trial showed that the control group's mean score was 5.97 ± 1.81, and the intervention group was 4.79 ± 1.96. Therefore, the sample size of the two study groups was 172 subjects (each group 86 cases) with an alpha of 0.05 level (type I error of 0.05, two-tail test) and *β* = 0.10 (a power of 90%). Considering a 20% dropout rate, we thought that increasing the sample size to 206 subjects was necessary to improve the validity of the study.

### 2.6. Measurement

Besides the patient's demographic data (age, gender, nationality, education, and marital state), the site of pain was recorded by data collectors as well. The primary outcome was measured by researchers at baseline (before performing the procedure, T0), 5 min (during the procedure, T1), and 5 min after treatment (postprocedure, T2) with FPS-R. It consisted of six facial expressions sorting pain levels (happy face = no pain and crying face = worst pain, ranging from 0 to 10), which was reliable and valid in older adults with dementia [25]. Secondary outcomes included three physiological parameters (blood pressure, heart rate, and oxygen saturation), any adverse reactions observed, satisfaction level from caregivers, acceptance of patients, and additional use of analgesics. Three physiological parameters were measured at T0, T1, and T2. Other secondary outcomes were recorded at T2. Caregivers' satisfaction level with this intervention was measured by a five-point scale (from 5 = very satisfied to 1 = very dissatisfied). The researcher assessed patients' acceptance by asking them if they would like to accept AA therapy once again. The adverse effects during the trial were recorded. Additional use of analgesics was documented during the procedure.

### 2.7. Statistical Analysis

All quantitative statistical analysis was conducted by SPSS 22.0 statistical software based on the intention-to-treat principle. Continuous data with normal distribution were analyzed by the independent sample *t*-test and were described as mean (standard deviation) or proportions. The chi-square test was used to analyze the ordinal categorical data. The repeated-measure analysis of variance was selected to describe three physiological parameters (heart rate, blood pressure, and O_2_ saturation) and the FPS-R scores. The Mann–Whitney test was used for the non-normal distribution parameters (satisfaction level of caregivers and acceptance of patients). Two-sided *p* value <0.05 was considered statistically significant.

## 3. Result

**Figure 1:**
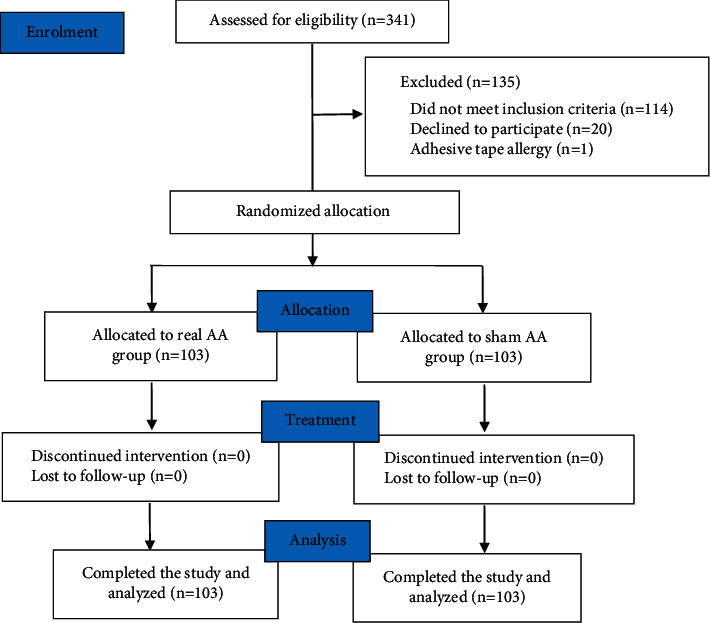
CONSORT study ﬂow diagram.

A total of 341 potential mild dementia patients with moderate-to-severe acute pain were screened for eligibility during the initial enrollment in three nursing homes in Yinchuan from April 2018 to March 2020, among which 135 dropped out, and 114 of them were further excluded as they did not meet the inclusion criteria. Subsequently, 20 were unwilling to participate in the study, and 1 was excluded owing to adhesive tape allergy. The 206 remaining patients completed the study, of which 103 patients were allocated in the real AA group and 103 in the sham AA group (Figure 1). The demographic and baseline characteristics of patients in the two groups were shown in Table 1. Patients' characteristics between the real AA group and sham AA group were well balanced in terms of age (82 (80–83) and 84 (83–85), *p*=0.24), gender (147 males, *p*=0.56), nationality (*p*=0.67), education background (*p*=0.08), marital state (*p*=0.58), occupation (*p*=0.55), the use of analgesic (*p*=0.61), and location of the pain (*p*=0.98). There were no statistically significant differences (*p* > 0.05).

**Figure 2:**
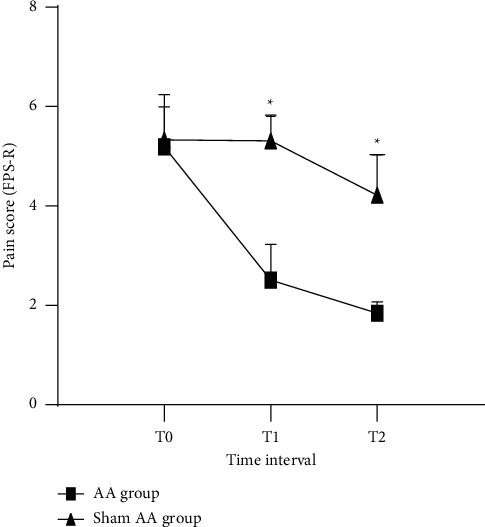
Pain score based on FPS-R for the AA and sham AA groups at T0, T1, and T2. APP = auricular point acupressure, FPS-R = face pain scale revised. T0: before performing the procedure, T1: at 5 min after implementing the procedure, and T2: after finishing the procedure. ^*∗*^Between groups, *p* < 0.01 (based on *t*-test).

Changes in pain scores based on the FPS-R for each group were summarized in Table 2 and Figure 2. There was a significant difference between the two groups (*p* < 0.01), but there was no statistical significance at T0. Pain scores in episodes treated with true AA decreased from 5.19 ± 0.81 (at T0) to 2.51 ± 0.72 (at T1) and 1.84 ± 0.23 (T2). For patients treated with sham AA, pain scores ranged 5.33 ± 0.91 from 5.31 ± 0.52 to 4.22 ± 0.81 at T0, T1, and T2, respectively.

The three physiological parameters were presented in Table 3. There was no difference between the two groups (*p* < 0.05). As summarized in Table 4, analysis of the additional use of analgesics found no significant difference between the two groups. Table 3 indicated that there was 3 (2.9%) patients having an additional use of painkillers in the sham AA group at T2, while it did not occur in the real AA group. No adverse events were found during the whole procedure between the two groups. The satisfaction level of caregivers in the sham AA group was significantly lower than the real AA group (*Z* = −11.653, *p* < 0.01; Table 5). This was also the case with the acceptance of patients for AA therapy (see Table 5). The acceptance of patients in the intervention group was notably higher than that of the control group (*Z* = −8.088, *p* < 0.01).

## 4. Discussion

In this present study, the real AA group has better results than the sham AA group in reducing pain intensity in dementia patients with acute pain. Furthermore, there are no side effects and complications associated with AA therapy.

Numerous studies have identified the safety and efficacy of AA therapy. The results of this study show that the use of AA is safe and effective for relieving acute pain, which was consistent with existing studies [17, 19]. A systematic review indicates that there is a significant difference in the AA group compared with the control group on acute postoperative pain after surgery relief [19]. Moreover, all of the 26 studies with 1,682 participants incorporated in the review did not observe adverse events, which recommends that AA be applied to control acute postoperative pain. In another study, Cha et al. showed that AA therapy is effective for alleviating primary dysmenorrhea of female high school students in South Korea (*t* = 32.187, *p* < 0.001) [17]. There is a study focusing on AA for decreasing the level of pain in elder patients with acute hip fractures. As a result, the patients in the true AA group have lower pain scores (*F* = 28, *p*=0.0001) than patients in the sham AA group, which demonstrates that AA is effective in reducing pain [26].

Statistical analysis of this study demonstrates that there is no significant difference among the three vital signs, including blood pressure, heart rate, and oxygen saturation. A randomized controlled trial evaluated the effect of the intervention group (AA group) on physiological indicators of variability in hypertensive patients compared with the control group. No differences are shown between the two groups in blood pressure and heart rate (*p* > 0.05) [27]. This study also displays that the satisfaction levels from caregivers and acceptance from participants in the AA group are higher than those in the control group. Other studies on AA have illustrated a similar result. Yeh et al. designed a feasibility study that AA is described as an adjunct analgesic to manage pain in cancer patients. The study found that AA was highly accepted by patients with cancer-related pain for AA as an adjunct analgesic [28]. Another randomized double-blinded study reported that patients in the real AA group had higher satisfaction than those in the sham AA group [17].

The Declaration of Montreal claims that access to pain management is a fundamental human right and the obligation of medical providers offering to a patient [29]. However, there are many studies focusing on the interventions in acute pain management, but a dearth of studies highlight the older patients. Even fewer studies have focused on acute pain management in nursing home residents with dementia. Abundant studies have reflected that optimal acute pain management in elderly patients with dementia remains an elusive goal [4, 8, 10, 13]. For dementia patients in the nursing home, it is recommended that a first-line treatment modality for pain is pharmacological intervention [11]. Considering the high-risk group of patients with senile dementia, drug safety (adverse effects and abuse potential) is highly concerned. Therefore, there is a need to explore a therapy that has the characteristics of non-invasive, has fewer side effects, and is effective for this population.

From the ancient record (*The Yellow Emperor's Classic of Internal Medicine* written in Chinese) to nowadays days, auricular therapy as a complementary and alternative technology has been employed for approximately 2,500 years. AA is one of the common methods of auricular therapy. And it was more widely applied for controlling various types of pain since the advent of Nogier's somatotopic maps. The ear is where 12 meridians are directly or indirectly connected. Stimulating it can achieve the purpose of qi-blood circulation, dredge meridians, and then analgesia according to traditional Chinese medicine (TCM) theory [18]. The mechanism of AA has a close relationship with the autonomic nervous system. The study has revealed that the analgesic effects of AA are caused by activation of the descending pain inhibitory pathways in the brain stem and thus ascending pain pathway inhibition. The application of AA activates the descending pain suppressing pathway along the spinal cord dorsal, thereby relieving pain. Hence, deep brain stimulation can exert a pain-relieving potency via inhibiting the dorsal lateral funiculus of the spinal cord [18]. In addition, benign stimuli generated by AA make the sympathetic nerve and parasympathetic nerve send impulses to the central nervous systems. After the integration of the system, the positive factors inside the human body are mobilized to raise the pain threshold [18, 30].

Many studies [18, 31] have shown that “shenmen” has the effect of stimulating and inhibiting the cerebral cortex. It was the main point for pain relief, suitable for various types of pain. “Subcortical” had the effect of sedation, analgesia, for clinical ear analgesia commonly used points. The function of the “adrenal gland” was to regulate the yin and yang of the human body and has analgesic and heat-clearing effects. The combination of the three acupoints strengthens the analgesic effect. In this study, they were elected as the primary acupoints for pain relief in the intervention group. However, their combination had no analgesic effect in the controlled group. So true AA worked more than sham AA.

Overall, the AA method has the undeniable advantages of safety, non-invasiveness (with less chance of infection), low cost, and even self-administration. Once AA tape is applied, it can stay in the auricular point for a long time. The patient can use his finger to gently press to stimulate the auricular points. AA seems to be a promising adjunct analgesic treatment for pain relief in nursing homes.

## 5. Limitations

The present study was designed to be single-blinded and performed in a single regional municipality. The pain level in the following hours after the procedure was not recorded in the study to evaluate the long-term effect (analgesic effect and safety). Furthermore, the study did not recruit different ages of patients, and our findings cannot be generalized to other patient settings. Thus, multiple regions and large-sample studies are warranted to determine the analgesic effect of AA therapy.

## 6. Conclusions

This study shows that real AA was an alternative analgesic modality in reducing acute pain in patients with mild dementia. There are few side effects during the study period. Medical providers may take the AA method into consideration for alleviating acute pain in nursing home residents with dementia, especially in low-resource settings.

## Figures and Tables

**Table 1:** Demographic characteristics of the two groups.

Variable	Group		*p*
	AA group (*n* = 103)	Sham AA group (*n* = 103)	
Age (years)^a^	81.1 ± 7.7	83.6 ± 5.3	0.243
Males^b^	71 (65.1%)	76 (70%)	0.561
Nationality^a^			
Han	90 (87.4%)	69 (67.0%)	0.829
Other	13 (12.6%)	34 (33.0%)	
Education^a^			
Primary school	6 (15.5%)	10 (9.7%)	0.076
Junior school	20 (19.4%)	16 (15.5%)	
High school	55 (53.4%)	59 (57.3%)	
Undergraduate	12 (11.6%)	18 (17.5%)	
Marital status^a^			
Single	1 (1.0%)	0 (0)	0.660
Married	23 (22.3%)	26 (25.2%)	
Divorced	6 (5.8%)	3 (2.9%)	
Widowed	73 (70.9%)	74 (71.8%)	
Occupation^a^			
Peasant	11 (10.7%)	16 (15.5%)	0.552
Worker	78 (75.7%)	76 (73.8%)	
Other	14 (13.6%)	11 (10.7%)	
Location of pain^a^			
Head or neck shoulder	10 (9.7%)	13 (11.9%)	0.979
Shoulder	5 (4.9%)	5 (4.9%)	
Upper limb	7 (6.8%)	4 (3.9%)	
Chest	1 (1.0%)	1 (1.0%)	
Abdomen	5 (4.9%)	7 (6.8%)	
Upper back	3 (2.9%)	3 (2.9%)	
Lower back	5 (4.9%)	7 (6.8%)	
Crotch	5 (4.9%)	7 (6.8%)	
Lower limbs	60 (58.3%)	55 (53.4%)	
Foot	2 (1.9%)	1 (1.0%)	
Pain scores before performing the procedure (FPS-R)^a^	5.19 ± 0.81	5.33 ± 0.91	0.705

Results are presented as means ± standard deviation and *n* (%). ^a^Based on chi-square test. ^b^Based on *t*-test.

**Table 2:** Pain score for the AA and sham AA groups at T0, T1, and T2.

Group	T0	T1	T2	Between groups
AA group	5.19 ± 0.81	2.51 ± 0.72	1.84 ± 0.23	*F* = 43.213
Sham AA group	5.33 ± 0.91	5.31 ± 0.52	4.22 ± 0.81	*p*=0.001
Time	*F* = 16.251,	*p*=0.001		
Group × time	*F* = 51.672	*p*=0.001		

AA: auricular acupressure, T0: before performing the procedure, T1: at 5 min after implementing the procedure, and T2: after finishing the procedure. Based on repeated measurement analysis of variance, *p* < 0.01.

**Table 3:** Vital signs at three time points (T0, T1, and T2).

Variable	Group		*p*
	AA group (*n* = 103)	Sham AA group (*n* = 103)	
SBp (mmHg)			
T0	140.06 ± 17.23	142.18 ± 15.45	0.131
T1	135.06 ± 15.06 9	142.47 ± 21.8	
T2	131.44 ± 14.60	136.16 ± 17.32	
DBp (mmHg)			
T0	77.96 ± 11.31	78.17 ± 9.69	0.296
T1	76.17 ± 10.356	77.21 ± 9.7	
T2	75.06 ± 10.28	77.84 ± 9.00	
HR (beats/min)			
T0	75.18 ± 10.58	72.38 ± 11.67	0.217
T1	73.52 ± 11.32	72.23 ± 11.45	
T2	73.63 ± 10.07	72.60 ± 10.67	
SpO_2_ (%)			
T0	95.81 ± 1.94	94.03 ± 1.20	0.089
T1	95.74 ± 2.11	94.72 ± 1.76	
T2	95.69 ± 1.85	95.02 ± 1.48	

AA: auricular acupressure, DBp: diastolic blood pressure, HR: heart rate, SBp: systolic blood pressure, SpO_2_: O_2_ saturation, T0: before performing the procedure, T1: at 5 min after implementing the procedure, and T2: after finishing the procedure. Based on repeated analysis of variance. No difference between groups, *p* > 0.05.

**Table 4:** Additional use of analgesics at T0, T1, and T2 between the two groups.

Group	T0	T1	T2	*p*
AA group (*n* = 103)				
Yes	0 (0)	0 (0)	0 (0)	0.246
No	103 (100%)	103 (100%)	103 (100%)	
Sham AA group (*n* = 103)				
Yes	0 (0)	0 (0)	3 (2.9%)	
No	103 (100%)	103 (100%)	100 (97.1%)	

Additional uses of analgesics: need to take analgesics for acute pain during the intervention. Based on Fisher's exact test. No significant difference between groups, *p* > 0.05.

**Table 5:** Satisfaction levels from caregivers and acceptance of patients.

Variable	*Z*	*p* ^*∗*^
Satisfaction from caregivers	−11.65	0.001
Acceptance of patients	−8.09	0.001

Based on Mann–Whitney test. ^*∗*^Statistically significant, *p* < 0.01.

## Data Availability

All data generated or analyzed during this study are included in this article. Further enquiries are available on reasonable request to the corresponding author.
